# Perception of emotionally loaded vocal expressions and its connection to responses to music. A cross-cultural investigation: Estonia, Finland, Sweden, Russia, and the USA

**DOI:** 10.3389/fpsyg.2013.00344

**Published:** 2013-06-21

**Authors:** Teija Waaramaa, Timo Leisiö

**Affiliations:** ^1^School of Communication Media and Theatre, University of TampereTampere, Finland; ^2^School of Social Sciences and Humanities, University of TampereTampere, Finland

**Keywords:** voice quality, expression, perception of emotions, valence, musical interests, cross-cultural

## Abstract

The present study focused on voice quality and the perception of the basic emotions from speech samples in cross-cultural conditions. It was examined whether voice quality, cultural, or language background, age, or gender were related to the identification of the emotions. Professional actors (n2) and actresses (n2) produced non-sense sentences (n32) and protracted vowels (n8) expressing the six basic emotions, interest, and a neutral emotional state. The impact of musical interests on the ability to distinguish between emotions or valence (on an axis positivity – neutrality – negativity) from voice samples was studied. Listening tests were conducted on location in five countries: Estonia, Finland, Russia, Sweden, and the USA with 50 randomly chosen participants (25 males and 25 females) in each country. The participants (total *N* = 250) completed a questionnaire eliciting their background information and musical interests. The responses in the listening test and the questionnaires were statistically analyzed. Voice quality parameters and the share of the emotions and valence identified correlated significantly with each other for both genders. The percentage of emotions and valence identified was clearly above the chance level in each of the five countries studied, however, the countries differed significantly from each other for the identified emotions and the gender of the speaker. The samples produced by females were identified significantly better than those produced by males. Listener's age was a significant variable. Only minor gender differences were found for the identification. Perceptual confusion in the listening test between emotions seemed to be dependent on their similar voice production types. Musical interests tended to have a positive effect on the identification of the emotions. The results also suggest that identifying emotions from speech samples may be easier for those listeners who share a similar language or cultural background with the speaker.

## Introduction

Basic emotions are thought to be universal in their manifestation since they are considered to be phylogenetic, evolutionary-survival related affects (Izard, 2007). The vocal expression and perception of these emotions tend to be based firstly on genetically inherited, and secondly on culturally learnt elements (Matsumoto et al., 2002). Also, the expression and perception of emotions expressed by music tends to be affected by both inherited characteristics and by cultural learning (Morrison and Demorest, 2009), and even by individual preferences, e.g., a piece of music may emotionally move one person but not another (Cross, 2001). In this paper it is hypothesized that the origin of speech and temporal experiences such as emotional and musical expressions are linked together in the evolution (Juslin and Laukka, 2003a). According to Richman (2001) “in the beginning speech and music making were one and the same: they were collective, real-time repetitions of formulaic sequences.” Moreover, Thompson et al. (2004) have suggested that “it seems unlikely that human evolution led to duplicate mechanisms for associating pitch and temporal cues with emotions.”

In voice research, voice quality is traditionally defined as the coloring of the speaker's voice (Laver, 1980), and in a narrower sense, as a combination of voice source (the air flow and vocal fold vibration), and filter functions (the vocal tract and formant frequencies) (Fant, 1970). The amount of subglottal air pressure and adduction of the vocal folds in the glottis determine the phonation type, whether it is hyperfunctional or hypofunctional. In a hyperfunctional phonation type the spectral slope is flatter and there is more energy and stronger overtones in the high frequency area than in a hypofunctional phonation type, where the slope is steeper and the overtones are weaker (Gauffin and Sundberg, 1989). Hyperfunctional phonation type is perceived as pressed voice quality and hypofunctional as breathy voice quality. Perceptual interpretations of the voice quality may either clarify or blur the meaning of the message, or change the whole information sent by a speaker.

Similarly to music, vocal expressions always have a fundamental frequency (F0) (excluding whisper), intensity (sound pressure level, SPL), and duration. These are the traditional parameters studied from the voice quality in emotional expressions. As sound is transmitted via vibrating objects there is no music without movement (Cross, 2001; Levitin and Tirovolas, 2009), and this connection between sound and movement tends to be evolutionarily based (Liberman, 1981; Liberman and Mattingly, 1985; Rizzolatti et al., 1996). As in voice production, the air pressure from the lungs makes the vocal folds vibrate, and without this action there is no vocal sound. According to the motor control theory and also the more recent theory of the mirror neurons speech is said to be understood rather in terms of its production than from the characteristics of the acoustic cues (Liberman, 1981; Liberman and Mattingly, 1985; Gentilucci and Corballis, 2006). In turn, the acoustic cues are connected to the physiological principles, and are the carriers of the emotional content of speech (see e.g., Juslin and Laukka, 2003b).

Human vocal communication inevitably conveys emotional messages – whether intended or not. Cultural differences do occur in humans in spite of the genetically based similarities in the expression and perception of the basic emotions (Matsumoto et al., 2002; Abelin, 2004). The cultural differentiation in music seems to occur by the end of the first year of life (Hannon and Trehub, 2005; Belin et al., 2011), and the cultural conventions of the music are learnt by the age of five (Trehub, 2003; Hannon and Trehub, 2005).

Typical of music, always based on harmonic relations between tones, are the rules (syntaxes) which govern the ways a tune is allowed to be composed. These rules are local and they deal with various alternating combinations of 1, 2, 3, 4, 5, or 6 tones (Leisiö and Ebeling, 2010). Typicality creates expectations and predictions of the characteristics of the musical sounds in a particular culture (Levitin and Tirovolas, 2009). However, the three basic elements of musical expression, frequency, intensity, and duration are not culture-specific as such.

There also appear to be similarities in the musical emotional expressions between cultures, e.g., emotional content of happy, sad, and fearful Western music has been reported to be recognized clearly above chance level by African listeners (Fritz et al., 2009). Balkwill and Thompson (1999) studied the perception of emotions in Western and Indian music and suggested that listeners are sensitive to unfamiliar tonal systems.

However, recognition of the emotions is more demanding in the absence of the familiar perceptual cues. This was also verified by Scherer et al. (2001), who conducted an extensive research project on the perception of vocal emotional utterances in seven countries, in Europe, Indonesia, and the USA. The vocal language-free portrayals used were produced by German professional actors, who expressed four emotions and a neutral emotional state. The emotions were perceived with 66% accuracy across countries. However, as the dissimilarities between the languages increased the accuracy of the perception decreased. As a result, the researchers stated that culture and language specific patterns may have an influence on the decoding processes of emotional vocal portrayals.

Sauter et al. (2009) studied perception of English and Himba non-verbal vocalizations representing basic emotions. Their results showed that listeners from both groups could identify the emotions, however, better accuracy was achieved when the producer and the listener were from the same culture.

Similar results were reported by Koeda et al. (2013) in a recent investigation of non-verbal “ah” affect bursts. The vocalizations were produced by French-Canadian actors. Canadian and Japanese participants served as listeners. It was found that the Canadian listeners recognized the emotions expressed, both positive and negative, more accurately than did the Japanese listeners.

Thompson et al. (2004), and Lima and Castro (2011) investigated whether music training assists speech prosody decoding. The researchers concluded that music training may facilitate the recognition of the emotional content of speech. Trimmer and Cuddy (2008) came to somewhat opposite conclusion. They reported that music training does not seem to be linked to the ability to recognize emotional speech prosody. Instead, emotional intelligence may predict sensitivity to emotion recognition from speech prosody, and this tends to require different processes than those required in musical or acoustical sensitivity. Strait et al. (2009) have stated that subcortical mechanisms are involved in the auditory processing of emotions, and musical training enhances these processes: training when younger than 7 years facilitates pitch and timber perception, and duration of training impacts processing of temporal features.

The present study was concerned with whether the voice quality of emotional speech samples affects the identification of emotions and emotional valence (on the axis positivity – neutrality – negativity). The second aim was to investigate cross-cultural perception, whether it is dependent on language or cultural background, age, or gender. Thirdly, whether the ability to recognize emotional states is related to musical interests was studied (Thompson et al., 2004; Trimmer and Cuddy, 2008; Levitin and Tirovolas, 2009; Strait et al., 2009). Therefore, the participants of the listening tests were asked on a questionnaire about their subjective musical interests. Listening tests for 250 randomly chosen, volunteer participants were conducted on location in five countries: Estonia, Finland, Russia, Sweden, and the USA.

## Materials and methods

### Acoustic and statistical analyses

Emotionally loaded sentences (n32) and protracted vowels [a:], [i:], [u:] (n8) were produced by Finnish professional actors (n2) and actresses (n2). They read aloud a non-sense text *(Elki neiku ko:tsa, fonta tegoa vi:fif:i askepan:a æspa. Fis:afi: te:ki sta:ku porkas talu.)* expressing six basic emotions, namely anger, disgust, fear, joy, sadness, surprise, and a neutral emotional state. These emotions were chosen since 4–6 of them (depending on the source) are thought to be universal (Murray and Arnott, 1993; Juslin and Laukka, 2003a; Mithen, 2006). Interest is sometimes also listed as one of the basic emotions since it is seen as the principle force in organizing consciousness and focusing attention (Izard, 2007, see also Scherer and Ellgring, 2007). Based on this definition, interest was included in the present investigation. The recordings were made by Sony Sound Forge 9.0 recording and editing system, and Rode NTK microphone at a professional recording studio MediaBeat in Tampere, Finland. The speakers' distance from the microphone was 40 cm. In the tests the listeners used Sennheiser HD 598 headphones.

Acoustic parameters were measured with Praat Software, version 5.2.18. A frequency range of 0–5 kHz and cross-correlation were used. F0, maximum pitch, SPL, filter characteristics (formant frequencies F1, F2, F3, F4), duration, mean harmonics-to-noise ratio (HNR, dB), number of pulses, and number and degree of voice breaks were measured. HNR measures perturbation in the voice signal. The number of voice breaks is the ratio between the number of pulse distances (min 1.25) and the pitch floor. Degree of voice breaks is the ratio between the non-voiced breaks and duration of the signal. (http://www.fon.hum.uva.nl/praat/manual/Voice_1__Voice_breaks.html.) The vowels were replayed consecutively to the participants in the listening tests. As the stress is always on the first syllable in Finnish language and thus carries the main communicational information, the acoustic parameters were studied only for the first [a:] vowel. Alpha ratio was calculated by subtracting the SPL in the range 50 Hz–1 kHz from SPL in the range 1–5 kHz (Frøkjær-Jensen and Prytz, 1973). Alpha ratio is used to get an illustration from the spectral energy distribution.

Emotional valence was coded by the researcher: positive valence (interest, joy and surprise) = 1, a neutral emotional state = 0, negative valence (anger, disgust, fear and sadness) = −1.

Statistical analyses were conducted using Excel and IBM SPSS Statistics 19 to investigate whether the voice parameters measured correlated with the identification of the emotions or valence and whether the perception of emotions differed by country, age, gender, or self-reported musical interests.

### Questionnaire and listening tests

Listening tests were conducted on location in five countries with different cultural and/or language backgrounds: Estonia, Finland, Russia, Sweden, and the USA. American English, Russian, and Swedish are related as members of the Indo-European linguistic family while Estonian and Finnish belong to the same Finno-Ugric language genus. As Nordic countries Finland and Sweden share a similar cultural background.

Fifty randomly chosen listeners in each country (25 males and 25 females × 5 countries = 250 listeners) participated in the perception test. The only criteria for participation was that the listeners were native speakers of the specific main language in each country, i.e., Estonian in Estonia, Finnish in Finland, Russian in Russia, Swedish in Sweden, and American English in the USA, and that the participants had lived most of their lives in the country. In Sweden, some of the listeners had one parent from another country, and one listener was adopted to Sweden as a baby, however, every listener spoke Swedish as their first language. The listeners were adults (18+ years old), mean age 33 years (Finland 47.5 years, Russia 34.5 years, Estonia 32 years, Sweden 27 years, and the USA 23 years).

The contact universities in the countries studied published the research project and called for volunteers to participate in the listening tests. Neither personal data registers nor invasive methods were used. All participants' anonymity was ensured. Consequently, no permission of the ethics committee was needed. The participants recruited in the USA were offered a course credit for participating.

The listening tests were conducted one by one with the listeners in an office (Finland and partly the USA), or in normal classroom conditions (Estonia, Russia, and partly the USA) or in a soundproof studio (Sweden). The researcher was alone with the listener in the test, except when a translator was needed in Russia. Listening tests are traditionally conducted in soundproof studio conditions. In the present study this was not required so as to be able to conduct the research independently using the facilities the universities in different countries were able to offer a visiting researcher. Furthermore, it was of interest to replicate the conditions of a normal social situation where people talk to each other having some random sounds around them, and nevertheless, focusing on listening to the speech and the voice of their interlocutors.

The participants completed a questionnaire eliciting background information, and responded to the following statements concerning their musical activities: (1) I like to listen to music. (2) It is easy for me to respond to music. (3) I am interested in singing. (4) I play a musical instrument. (5) I am interested in dancing. (6) It is easy for me to dance in the correct rhythm. (7) It is easy for me to learn a new melody. (8) Music may affect my mood. (9) Music may cause me physical reactions. The idea was to study the participants' subjective opinion about their relation to music, not to measure their activity or education in music.

The questionnaire and the emotion labels were translated by university teachers, either native speakers of the language (Estonian and English) or Finnish teachers in Swedish and Russian.

In the perception test the listener first heard four two-sentence non-sense samples, one from each speaker, and then one example of each emotion expressed by the four speakers. The researcher named the samples by the emotion before replaying them one by one in order to familiarize the listener with the speakers' voices and the vocal variation the speakers used in the emotional expressions. Next, the researcher replayed the 32 emotional nonsense sentences one by one (eight emotions × four speakers), and the listener reported orally which emotion he/she perceived. The researcher wrote down the answers given. Finally, the listener heard eight simple protracted vowel samples, two emotions from each speaker, and chose his/her answer again from the list of the eight emotions expressed. Free choice was not used. The test took about 35 min for each listener.

All the samples were replayed in the same random order from the researcher's computer to the participants. The listeners did not have to use any equipment while listening and answering. In unclear cases the participants were instructed to choose the nearest emotion to what they assumed to be the target. They were asked to choose neutral only when they thought there was no particular emotion expressed. The participants were instructed to answer as briefly as possible. On the other hand they were allowed to listen to a sample as many times as they felt they needed to (usually 1–2 times). They were also allowed to listen to the previous samples again so as to avoid possible order effects.

## Results

### Voice quality

In vowel [a:] alpha ratio correlated significantly negatively with duration in both genders. In the sentences alpha ratio and SPL correlated significantly positively. Alpha ratio and SPL have been shown to vary together (Nordenberg and Sundberg, 2003; Sundberg and Nordenberg, 2006). Duration correlated negatively with F0. These results suggest that in hypofunctionally produced samples duration is longer than in hyperfunctional produced samples.

Significant correlations with share of identified *emotions* and voice parameters were found in both genders for mean HNR, number of voice breaks and SPL, and in females also for maximum pitch and number of pulses. Significant correlations with share of *valence* and voice parameters identified were found in both genders for number of pulses and number of voice breaks, and in males also for duration (Table 1).

**Table 1:** **Significant results for Pearson correlation between voice quality parameters and the share of identified emotions and valence (*p* < 0.05)**.

***r*-values**	**Max pitch**	**N pulses**	**N voice breaks**	**HNR (dB)**	**Duration**	**SPL (dB)**
**SIGNIFICANT CORRELATIONS OF VOICE PARAMETERS WITH SHARE OF IDENTIFIED EMOTIONS AND VALENCE**						
Identified emotions, male listeners	ns	ns	0.427	0.44	ns	-0.356
Identified emotions, female listeners	0.391	0.342	0.448	0.462	ns	-0.314
Identified valence, male listeners	ns	0.395	0.385	ns	0.353	ns
Identified valence, female listeners	ns	0.334	0.344	ns	ns	ns

Number of voice breaks was highest for sadness and lowest for anger, and degree of voice breaks was highest for fear and lowest for joy. The voice production type in sadness and fear tends to be more hypofunctional than in anger and joy thus, having less energy e in the higher frequency area of the spectrum.

The mean duration of the sentence samples was 9652 ms, and vowels 930 ms. Anger in males, and joy in females had the lowest durations for the sentences. Negative emotion of sadness followed by fear had the longest durations in both genders.

### Questionnaire

Degree of tiredness or mood tended to be non-significant features in relation to the identification accuracy of the emotional samples. Seventeen participants reported impaired hearing (Estonia 1, Finland 8, Russia 2, Sweden 5, and the USA 1).

The results of the Student's *T* test showed that those who reported impaired hearing did not identify the emotions less successfully (69% identified) than those with normal hearing (70% identified).

The listeners were divided into two groups, under 40 years and 40+ years in order to study perceptual age differences. The younger group identified emotions with 70% accuracy and valence with 91% accuracy, and the older group emotions with 68% and valence with 90% accuracy. When Pearson correlation was studied by country, a slight negative correlation between age and the identification of the emotions was found for Finland, Russia, and the USA (Table 2).

**Table 2:** **Percentages and significance for identified emotions and valence by age and country**.

**Country**	**Gender**	**Age (years)**	**Identification %**	
			**Emotion (%)**	**Valence (%)**
**IDENTIFIED EMOTIONS AND VALENCE BY AGE AND COUNTRIES**				
Estonia	Male	<40	71	94
		40+	77	94
	Female	<40	70	92
		40+	73	92
Finland^*^	Male	<40	82	96
		40+	74	94
	Female	<40	81	97
		40+	78	96
Russia^*^	Male	<40	65	89
		40+	54	84
	Female	<40	64	85
		40+	61	86
Sweden	Male	<40	69	89
		40+	66	89
	Female	<40	73	91
		40+	68	83
USA^*^	Male	<40	64	88
		40+	48	77
	Female	<40	61	86
		40+	.%	.%

The results are presented for the first four samples, sentences and vowels (There were no female listeners 40+ years in the USA).

* Significant negative correlation with age: Finland r = −0.333, Russia r = −0.350, USA r = −0.302.

The first statement in the questionnaire was “I like to listen to music.” By this statement the idea was to measure the degree of consumption of music. The results showed that the degree of consumption by listening to the music did not seem to be associated with the emotions or valence identified in the vocal samples (Table 3).

**Table 3:** **The results of the 250 questionnaires from the five countries studied and their relation to the identified emotions**.

**“Yes” answers (%)**	**Estonia (%)**	**Finland (%)**	**Russia (%)**	**Sweden (%)**	**USA (%)**	**Total (%)**	**Sig.**
**RESULTS OF THE QUESTIONNAIRES BY COUNTRY AND THEIR RELATION TO THE EMOTIONAL SAMPLES IDENTIFIED**							
I like to listen to music	90	92	90	96	100	94	ns
It is easy for me to respond to the music	66	78	36	68	78	65	***
I am interested in singing	24	28	10	48	18	26	***
I play a musical instrument	24	24	22	42	14	25	*
I am interested in dancing	16	18	6	4	44	18	***
It is easy for me to dance in the correct rhythm	70	66	40	67	32	55	***
It is easy for me to learn a new melody	48	58	38	60	30	47	**
Music may affect my mood	70	100	70	92	76	82	***
Music may cause me physical reactions	30	62	62	82	32	54	***

The percentages are the “Yes” answers to the statements. “Yes” answers and the identification of the emotions and valence were significantly associated (excluding the first statement). Significance of the relationship appears on the far right.

Statements:

*** p < 0.001,

** p < 0.01,

* p < 0.05, ns) non-significant in independent samples.

Student's T test for equality of means.

The other statements concerning musical interests were statistically significantly associated with the emotions and valence identified. Those participants who reported engaging in musical interests and responding to music were compared to those who did not have a clear response to these activities. It was found that the listeners reportedly engaging in music differed significantly in the share of the identified emotions and valence from the listeners who did not report musical interests or sensitive response to music (Table 3).

Females reported significantly more often than males being interested in singing while males reported playing a musical instrument significantly more often than females. When studied by country, those who were interested in singing and who played a musical instrument were most often Swedish listeners. “I am interested in dancing” was most often answered “Yes” by the US listeners.

Emotional states of fear, interest, and joy were most frequently associated with musical interests. Neutrality was not associated with any of the musical interests. “It is easy for me to learn a new melody” and “I am interested in singing.” were the statements which seemed to be engaged with most of the identified emotions. The statement “It is easy for me to dance in the correct rhythm” was not emotion specific and was not associated with any particular emotion (Table 4).

**Table 4:** **Emotions significantly associated with musical interests**.

**MUSICAL ACTIVITIES ASSOCIATED WITH IDENTIFICATION OF EMOTIONS**		
I like to listen to music			Sadness^*^
1 Yes	2 No	3 Only in the background	
It is easy for me to respond to the music			Fear^*^
1 Yes	2 No	3 Sometimes	
I am interested in singing			Anger^***^, disgust^***^, fear^*^, interest^*^
1 Yes	2 No	3 Not in public	
I play a musical instrument			Fear^**^, interest^*^, joy^*^
1 Yes	2 No	3 Not any more	
I am interested in dancing			Fear^**^, interest^**^
1 Yes	2 No	3 Not in public	
It is easy for me to dance in the correct rhythm			Ns
1 Yes	2 No	3 Sometimes	
It is easy for me to learn a new melody			Anger^**^, disgust^*^, fear^**^, interest^*^, joy^*^, surprise^*^
1 Yes	2 No	3 Sometimes	
Music may affect my mood			Fear^***^, interest^*^, joy^**^
1 Yes	2 No	3 Sometimes	
Music may cause me physical reactions			Fear^*^, interest^*^, joy^*^
1 Yes	2 No	3 Sometimes	

Statements:

*** p < 0.001,

** p < 0.01,

* p < 0.05, ns.) non-significant in independent samples.

Student's T test for equality of means.

### Listening tests

**Figure 1:**
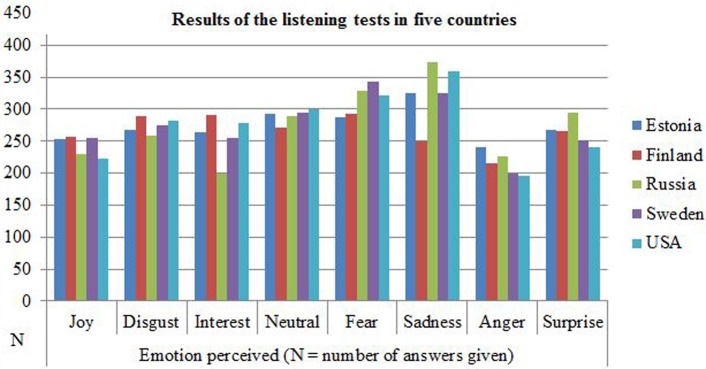
**Results of the listening test by emotion and country including the first four samples, the sentences and the vowels**.

Crohnbach's alpha for the listening test by country was: Finland 0.945, Estonia 0.929, Sweden 0.905, the USA 0.874, and Russia 0.871. The results showed that the percentage of emotions and valence identified was clearly above the chance level in each of the five countries with different language and/or cultural backgrounds. A confusion matrix in percentages and numbers for the emotions identified is shown in Table 5. Sadness and fear were the most frequently chosen emotional states for an answer, followed by neutrality. Anger was the most rarely chosen answer (Figure 1).

**Table 5:** **The line “Count” in the confusion matrix of the emotions expressed and emotions perceived shows the numbers of answers given**.

**Integer = *N***				**Emotion expressed**					
**Emotion perceived**		**Joy**	**Disgust**	**Interest**	**Neutral**	**Fear**	**Sadness**	**Anger**	**Surprise**	**Total**
**EMOTION PERCEIVED × EMOTION EXPRESSED CROSSTABULATION**										
Joy	Count	**952**	11	61	2	6	0	7	178	1217
	% within emotion perceived	78.2%	0.9%	5%	0.2%	0.5%	0%	0.6%	14.6%	100%
	% within emotion expressed	64.2%	0.7%	4.9%	0.2%	0.4%	0%	0.6%	14.2%	11.1%
Disgust	Count	45	**846**	3	37	62	35	293	51	1372
	% within emotion perceived	3.3%	61.7%	0.2%	2.7%	4.5%	2.6%	21.4%	3.7%	100%
	% within emotion expressed	3%	57%	0.2%	3%	4.2%	2.4%	23.4%	4.1%	12.6%
Interest	Count	128	25	**742**	26	41	3	77	246	1288
	% within emotion perceived	9.9%	1.9%	57.6%	2%	3.2%	0.2%	6%	19.1%	100%
	% within emotion expressed	8.6%	1.7%	59.4%	2.1%	2.8%	0.2%	6.2%	19.7%	11.8%
Neutral	Count	38	53	81	**1106**	37	17	106	10	1448
	% within emotion perceived	2.6%	3.7%	5.6%	76.4%	2.6%	1.2%	7.3%	0.7%	100%
	% within emotion expressed	2.6%	3.6%	6.5%	88.5%	2.5%	1.1%	8.5%	0.8%	13.2%
Fear	Count	84	93	17	12	**1174**	154	24	16	1574
	% within emotion perceived	5.3%	5.9%	1.1%	0.8%	74.6%	9.8%	1.5%	1%	100%
	% within emotion expressed	5.7%	6.3%	1.4%	1%	79.2%	10.4%	1.9%	1.3%	14.4%
Sadness	Count	28	192	16	49	75	**1266**	7	1	1634
	% within emotion perceived	1.7%	11.8%	1%	3%	4.6%	77.5%	0.4%	0.1%	100%
	% within emotion expressed	1.9%	12.9%	1.3%	3.9%	5.1%	85.4%	0.6%	0.1%	14.9%
Anger	Count	21	249	4	18	36	6	**708**	37	1079
	% within emotion perceived	1.9%	23.1%	0.4%	1.7%	3.3%	0.6%	65.6%	3.4%	100%
	% within emotion expressed	1.4%	16.8%	0.3%	1.4%	2.4%	0.4%	56.6%	3%	9.9%
Surprise	Count	187	14	326	0	52	2	28	**711**	1320
	% within emotion perceived	14.2%	1.1%	24.7%	0%	3.9%	0.2%	2.1%	53.9%	100%
	% within emotion expressed	12.6%	0.9%	26.1%	0%	3.5%	0.1%	2.2%	56.9%	12.1%
Total	Count	1483	1483	1250	1250	1483	1483	1250	1250	**10932**
	% within emotion perceived	13.6%	13.6%	11.4%	11.4%	13.6%	13.6%	11.4%	11.4%	100%
	% within emotion expressed	100%	100%	100%	100%	100%	100%	100%	100%	100%

The integers presented in bold face are the emotions identified in numbers. The other integers on the “Count” line show the numbers of confusions with the other emotions. The line “% within emotion perceived” shows the percentage of the answers given for each emotion. The line “% within emotion expressed” shows the percentage for the identification of the emotion in question.

For the first four samples the percentage of identified emotions was 59% and valence 87%, for the sentences 70% and valence 90%, and for the vowels 69 and 90% respectively. The result for the first four samples was from the 233 participants since the first 17 Finnish listeners missed these samples at the beginning of the present research project. As the accuracy percentage of identification was higher for the sentences than for the first four samples it may be assumed that familiarizing the listeners with the variations of the speakers' voices may have improved their recognition of the target emotions. The familiarizing did not seem to affect the recognition as much of emotional valence which was fairly high already before the familiarizing. Negative emotions were identified slightly more accurately than positive ones.

The younger listeners identified sadness significantly better than the older listeners (*p* = 0.036), who identified joy (*p* = 0.021), surprise (*p* = 0.002), and neutrality (*p* = 0.024) significantly better than the younger ones.

The binomial test conducted on the samples showed that 10 samples were identified with under 50% accuracy: two from the first four samples, disgust (24%), and fear (45% accuracy), from the sentences two samples of anger (13%, 31%), disgust (38%), joy (26%) interest (44%) and surprise (43%), and from the vowels joy (36%) and surprise (42% accuracy). Seven of these samples were produced by male speakers.

A number of confusions of the emotions perceived occurred in the listening test. Hypofunctionally produced emotions of sadness and fear were frequently confused with one another, likewise the hyperfunctionally produced negative emotions of anger and disgust. On the other hand, disgust was also confused with sadness by the listeners in Russia, Sweden, and the USA but not in Estonia or Finland. Positive emotions of joy, surprise, and interest were confused with one another, and thus the percentage for their identification was relatively low.

There was a tendency in the perception test that the more similar the listeners' language or cultural background was to those of the speakers', the more accurate the emotion recognition was, and conversely, the more different the language or cultural background was the less accurate the emotion recognition was. The quartiles studied by country showed that 1/4 of the listeners, e.g., in Estonia identified 55%, 1/2 identified 68%, and 1/4 at least 77% of the emotion samples. Variation was widest for Finland. The percentages fall into the quartiles roughly similarly for Estonia and Sweden, and for Russia and the USA. Finnish listeners were most accurate in the identification (Table 6).

**Table 6:** **The quartiles for the shares of identified emotions studied by country**.

**LISTENER QUARTILES BY COUNTRY**			
% = Share of identified emotions			
Listener quartiles	25	50	75
Estonia (%)	55	68	77
Finland (%)	41	76	85
Russia (%)	52	59	68
Sweden (%)	55	66	77
USA (%)	50	59	67

The logistic regression model (Response = Emotion identified/not identified) showed that the five countries perceived the emotions expressed significantly differently. The identification was connected to the age of the listener. The interaction effect of the speaker gender, country and emotion expressed was significant. The greatest difference between the emotion identification and the gender of the speaker was found for Estonia and Russia. There, most of the non-identified samples were produced by males. Listener's gender was non-significant (Table 7).

**Table 7:** **Test of model effect in the logistic regression model of the combined effects on the identification of the emotions**.

**Main effects + all significant (p < 0.05) 2-way and 3-way effects**	**Type III Wald df Sig. Chi-Square**		
**Source**			
**TESTS OF MODEL EFFECTS**			
(Intercept)	366.2	1	0.000
Speaker gender	323.9	1	0.000
Listener gender	1.00	1	0.32
Country	113.5	4	0.000
Emotion expressed	629.6	7	0.000
Age	19.3	1	0.000
Speaker gender × country	12.3	4	0.015
Speaker gender × emotion expressed	204.9	7	0.000
Country × emotion expressed	103	28	0.000
Speaker gender × country × emotion expressed	97.6	28	0.000

When studied by country, gender differences were found for only two countries: Estonian males recognized the valence of the first four samples significantly better than did the Estonian females. Swedish males recognized emotions from the sentences significantly better than did Swedish females. However, the differences between genders did not vary significantly among all five countries (Table 8).

**Table 8:** **Accuracy of the identification of the emotions and valence in percentages when studied by country and gender**.

		**Male speakers**		**Females speakers**	
		**Emotions (%)**	**Valence (%)**	**Emotions (%)**	**Valence (%)**
**EMOTION AND EMOTIONALVALENCE IDENTIFIED BY COUNTRY**					
Finland	Male listeners	70	93	82	95
	Female listeners	73	96	85	96
Estonia	Male listeners	63	91	82	97
	Female listeners	66	90	76	95
Sweden	Male listeners	64	86	73	93
	Female listeners	68	87	77	93
USA	Male listeners	59	84	66	91
	Female listeners	55	83	68	90
Russia	Male listeners	54	82	69	93
	Female listeners	54	79	71	91

The emotions produced by males were perceived with 62% accuracy and valence with 87% accuracy, those produced by females corresponding with 74 and 94% accuracy. The difference was statistically significant (Table 9).

**Table 9:** **Results of the emotionally loaded samples identified in percentages by gender of speakers and listeners**.

**Listeners**	**Target matching vocal samples**			
	**Male speakers**		**Female speakers**	
	**Emotions (%)**	**Valence (%)**	**Emotions (%)**	**Valence (%)**
Males	62	87	74	94
Females	63	87	75	94

## Discussion

### Voice quality

Identification of valence in both genders appeared to be connected to the number of pulses and number of voice breaks. In a hyper-functional voice quality (e.g., in joy and anger) number of pulses is higher per time-domain than in a hypofunctional voice production type (e.g., Waaramaa et al., 2006). Highest number of voice breaks was found for sadness, and highest degree of voice breaks for fear which were both hypofunctionally produced utterances. Voice breaks and perturbation of voice signal tended to be discriminating features connected to the pressed/breathy voice quality in the emotional utterances.

The results suggest that valence is more important in the perception process of the vocal expressions and is therefore of greater communicative importance than the actual emotions. It was shown in a recent study by Waaramaa and Kankare (2012) that statistically significant differences between valences were already found on micro level emotional expressions which were calculated from the electroglottogram (EGG) signal. EGG was used to measure the contact quotient (CQ_EGG_) of the vocal folds. When the vocal folds were 25% closed (25% threshold level) significant differences were already found between valences for the CQ_EGG_. Significant gender differences have been found at the 55% threshold level (Higgins and Schulte, 2002). Consequently, differences between emotions may occur only on higher threshold levels, i.e., later in the expression. Glottal behavior has likewise been reported to affect valence perception by Laukkanen et al. (1997) and (Waaramaa et al., 2008, 2010).) Thus, from the communicative perspective, expression of valence seems to precede the expression of gender or the actual emotion in speech samples.

Formant frequencies measured in vowel [a:] did not show significant differences between emotions in the present material. Nor was it expected for F1 and F2, since they are determined by the vowel expressed. Instead, in earlier investigations F3 and F4 have shown higher frequencies in positive emotions than in negative ones (Waaramaa-Mäki-Kulmala, 2009). This was also the case in the present material, but not significant. Waaramaa et al. (2006) studied synthesized vowel [a:] samples with raised, lowered and removed third formant frequency (F3) and valence perception from the samples. The results showed that the raised F3 frequency was perceived more often as positive than the other samples. It was concluded that samples with sufficient energy in the high frequency area of F3 may affect perception of positive valence from a signal.

However, it has been suggested by Laukkanen et al. (2008) that at least valence – if not actual emotions – can be perceived from emotional expressions even with several vocal cues eliminated (see also Waaramaa et al., 2006). This concurs with the idea of motor control and mirror neuron theory that speech can be understood rather in terms of its production than from the characteristics of the acoustic cues (see Introduction in this paper). Thus, general acoustic patterns for emotions can be only roughly presented.

### Questionnaire

Language differences emerged when the original Finnish questionnaire was translated into Russian and Swedish. It occurred that the statement “It is easy for me to respond to music.” was translated into Russian in such a way that the grammatical subject (me) was changed into the object (on me): “Music has a strong effect on me.” It can be speculated whether this has had an effect on the answering to this statement since the percentage of the “Yes” answers was about 50% less in Russia than in the other countries. Another problem with the translation occurred when the Finnish word for “anger,” *viha* was translated into Swedish as *hat*, “hate” instead of its correct equivalent of *ilska*, “ill temper,” “anger.” This problem was explained to the last 1/4 of the participants in the listening test in Sweden.

The statements “It is easy for me to learn a new melody” and “I am interested in singing” were connected to most of the identified emotions. This may partly refer to the underlying intonation of the speech (melody recognition) and partly to the similarities of vocally produced utterances recognized by those who were interested in singing which is also a form of vocal expressions.

Most of those listeners who were interested in singing and who played a musical instrument were Swedish listeners. This result may be affected by the fact that the listening tests were conducted with help from the Music Acoustics Group at KTH, Royal Institute of Technology in Stockholm. Thus, many of the participants were involved with music through their professions, studies or hobbies. In this respect, the participants in the other countries studied may have been more heterogeneous than those in Sweden.

### Listening test

The results of the present study showed that the percentage of the emotion identification and valence was clearly above the chance level in each of the five countries with different language and/or cultural backgrounds. Gender had no role in the perception of emotions or valence between the five countries studied. This result concurs with the findings by Koeda et al. (2013). Yet individual differences may be significant.

The speakers of the voice samples spoke Finnish as their native language, hence they read the non-sense text aloud using the Finnish prosody. This may be the reason why the Finnish listeners scored highest on the identified samples. A similar result was reported by Scherer et al. (2001) and by Abelin and Allwood (2000). Matsumoto et al. (2002) and Abelin (2004) have suggested that interpretation of prosody is easier for native speakers of the language in question. Abelin (2004) also has stated that the prosody of emotional expression is always related to the particular language spoken, and never occurs in isolation (see also Iversen et al., 2008). Thus, the Finnish listeners were at an advantage in the perception test as they obviously recognized the prosody more easily than the other listeners in the other countries, and could connect the prosody to the linguistic expressions even without meaningful words used. Finnish listeners perceived most rarely neutrality and most frequently joy and interest – but also disgust when compared to the other countries.

In their earlier study Schirmer and Kotz (2002) used event-related potentials (ERP) to study how their participants judged the valence of the prosody of a German verb and the emotional meaning of the word.

Interaction between emotional prosody and word meaning was found in females but not in males. Males appeared to process the meaning and the emotional prosody independently of each other. The researchers also argued that females are faster and more accurate in judging emotional information than males (Schirmer and Kotz, 2002; Schirmer et al., 2002, see also Besson et al., 2002; Imaizumi et al., 2004; Fecteau et al., 2005; Schirmer and Simpson, 2008). In the present investigation non-sense utterances were used. Thus there was no meaning in the words. However, gender differences were not studied here by ERP, consequently, it can only be stated that no gender differences in the accuracy of the emotion or valence perception were found. This concurs with the findings combining brain evolution, gender differences, and music (Falk, 2000).

The perceptual confusion of the three positive emotions interest, joy, and surprise may indicate that from the evolutionary-survival perspective it may not have been crucial to distinguish between these emotions. The emotional state of joy was poorly recognized Scherer et al. (2001) have reported similar results for joy Sauter et al. (2009) have stated that communication of positive emotions may be restricted to the members of the same social or cultural group and function as consolidation of that group.

Identification of anger was not particularly accurate in the present study. This may be in part due to the chosen expression types by the speakers. They tended to express more cold anger than hot anger or rage. Hot anger is undoubtedly easier to identify than cold anger. One reason for not using hot anger was that the expressions had to meet the quality criteria set by the software programs in order to conduct the acoustic analyses. Further, perception of anger (Ekman, 2004; Abelin, 2008a,b) and disgust (Banse and Scherer, 1996) may be more dominated by the visual than auditive information. However, the negative emotions of anger and disgust have been reported to be confused in visual perception tests as well (Matsumoto et al., 2002). Matsumoto et al. have suggested that the semantics of these emotions is similar and they share the elicitors of the emotion. Also, it may be easier to distinguish between positive and negative emotions (i.e., to identify valence) than between emotions which share the same valence, e.g., two negative emotions (Thompson et al., 2004). Moreover, Koeda et al. (2013) have reported significant cross-cultural differences in the perception of anger, disgust, and fear.

In the present study, the emotional state of fear tended to be well recognized from the auditive characteristics (see also, Abelin, 2008a,b). However, fear was frequently confused with sadness, obviously due to the similarities in their acoustic cues and the large number of voice breaks they shared. These negative emotions tended to be more irregularly expressed than the positive emotions (see also Juslin and Laukka, 2003a). Accordingly, Kotlyar and Morozov, 1976, see also Scherer, 1995) have reported longer pauses between syllables and shorter syllable duration for fear than for the other emotions in the European opera singing tradition they studied. The confusion of sadness and fear concurs with the results of an earlier study by Scherer et al. (2001). Nevertheless, sadness and fear were well recognized: the two emotions together yielded 82% accuracy and valence 94% accuracy.

Laukka and Juslin (2007) and Lima and Castro (2011) have stated that recognition, especially of negative emotions, tends begin to change during middle age. In the present study a negative correlation was found between age and the emotions identified for Finland, Russia, and the USA. Young listeners have been reported to be more accurate than older listeners at recognizing disgust, fear and anger from speech samples (ibid.). This was also seen in the present results. Negative emotion of sadness was significantly better recognized by young listeners, and positive emotions of joy and surprise, and additionally neutrality were significantly better recognized by old listeners. Moreover, the US participants were the youngest listeners and they chose disgust most frequently as an answer to the sentences.

From the evolutionary-survival and reproduction viewpoints it may be important for young people to be able to recognize negative emotions. Additionally, sadness may be an emotion which strengthens the bond between the members of the community. An accurate identification of positive emotions may imply older people's higher tolerance or understanding for the less serious features.

As some of the US listeners were offered course credit for participating in the present test, it may be speculated whether they were completely volunteers or not, and if on the one hand the willingness, or on the other hand the advantage gained, was the “real” motive for participating. Either way, it may have had an effect on the US results.

Even though the speakers were professionals, significant differences occurred in the perception of emotions expressed. It must be stressed that the samples produced by one actress were easiest to recognize throughout the countries, and this may explain the bias in the results of the perception. Coincidentally, somewhat problematic differences in the vocal samples used have also been reported previously (Scherer et al., 1991). Speaker gender has previously been reported to have a significant effect on the identification of emotions (Koeda et al., 2013). Several studies of the vocal characteristics of emotional expressions have also shown that individual differences are significant (e.g., Ladd et al., 1985).

Whether actor portrayals should or should not be used in emotion research has frequently been discussed. Utterances produced by actors are claimed to be stereotypical and controlled, not genuine expressions. However, in such claims genuine is never defined. This raises another question about how genuine (or pure) our emotions are in “real life” as they are mixed in our minds with other ongoing emotions quite randomly and individually (see Izard, 2007). Do we know how a pure single emotion always needs to be manifested by all humans? However, the emotional samples of the present study were fairly well recognized by the listeners. Thus, there must have been some cues, either universal or cultural, which the listeners thought they recognized as expressing the specific emotional states. A number of authorities, cultural, and social systems control and regulate our social and emotional behavior, competence, and skills (Banse and Scherer, 1996; Sauter et al., 2009). To have social competence or skills requires subjective control. Thus, it does not seem reasonable to claim that in “real life” emotions are uncontrolled and hence, “genuine.” It seems rather that in “real social life” emotional expressions are restricted and socialized to fit the commonly accepted norms, rules, and limits of the particular society. Consequently, it may sometimes be difficult to interpret the emotional message if the verbal and non-verbal signals are ambiguous. The expressions produced by an actor may thus be more simple and clear as he only uses those vocal cues which are necessary to convey the target emotion. This in turn, may lack realistic situational constraints (Scherer and Ellgring, 2007).

### Vocal emotions and music

Humans tend to remember better the general structure of the melody line, i.e., the contour than the exact sizes of individual intervals between tones (Levitin and Tirovolas, 2010). The prosodic contour of an utterance may underlie the significance of a musical phrase or proto-musical behavior (Cross, 2001). According to Panksepp (2009/2010) it is possible that without prosodic pre-adaptations from evolving humans music might never have emerged. Juslin and Laukka (2003a) have suggested that the emotional expressiveness of music is based on the similarities of the emotional acoustic cues in vocal expressions. Hence, emotional music and speech may engage the same neural processes (Juslin and Västfäll, 2008).

In the present investigation, the positive emotions were expressed with fewer voice breaks and in a more rhythmical manner than the negative emotions. Speaking in a friendly manner has been shown to carry more melodic characteristics than speaking in an unfriendly way (Fónagy, 1981). Motherese, the speech directed to babies is also melodic and rhythmic (Trehub, 2003). Melodicity has suggested to be a third dimension apart from pitch and time. Melodicity is defined as “the perceptual response to the higher or lower degree of regularity/continuity/predictability of the fundamental frequency curve within each syllable” (Fónagy, 1981). Melodicity can also be used as a means in identifying the emotion. One male listener in the present study explained how he perceived the emotional samples as melodies and based on the melody he decided which emotion he heard. His identification was exceptionally accurate.

## Conclusion

Identification of emotions from speech samples tended to be affected by voice quality and by a similar language and/or cultural background. Hence, vocal non-verbal communication affects interpretation of emotions even in the absence of language. It tends to be interpreted differently by speakers of different languages. Musical interests facilitate distinguishing between emotions.

Finally, it has to be stated that all the five countries studied are culturally relatively close to each other. In the future study a clearly different culture representing a totally different language background should be included in the comparison of the countries. This culture and language will be Arabic in Egypt.

### Conflict of interest statement

The authors declare that the research was conducted in the absence of any commercial or financial relationships that could be construed as a potential conflict of interest.

## Appendix

**Table:**

1	Name	——————————————–		
2	Age	——————————		
3	Gender	1 Male	2 Female	
4	My hearing is	1 Normal	2 Impaired	
5	At the moment I am	1 Alert	2 Tired	3 In between these two
6	At the moment my mood is	1 Good	2 Bad	3 Neutral
7	I like to listen to music	1 Yes	2 No	3 Only in the background
8	It is easy for me to respond to the music	1 Yes	2 No	3 Sometimes
9	I am interested in singing	1 Yes	2 No	3 Not in public
10	I play a musical instrument	1 Yes	2 No	3 Not any more
11	I am interested in dancing	1 Yes	2 No	3 Not in public
12	It is easy for me to dance in the correct rhythm	1 Yes	2 No	3 Sometimes
13	It is easy for me to learn a new melody	1 Yes	2 No	3 Sometimes
14	Music may affect my mood	1 Yes	2 No	3 Sometimes
15	Music may cause me physical reactions	1 Yes	2 No	3 Sometimes

### Please choose from these emotions which of them you hear:

**Table:**

1	Neutral
2	Sadness
3	Fear
4	Anger
5	Disgust
6	Joy
7	Surprise
8	Interest
